# Genome-Wide Identification, Characterization, and Expression Profile Analysis of *CONSTANS-like* Genes in Woodland Strawberry (*Fragaria vesca*)

**DOI:** 10.3389/fpls.2022.931721

**Published:** 2022-07-12

**Authors:** Xinyong Zhao, Fuhai Yu, Qing Guo, Yu Wang, Zhihong Zhang, Yuexue Liu

**Affiliations:** ^1^College of Horticulture, Shenyang Agricultural University, Shenyang, China; ^2^Liaoning Key Laboratory of Strawberry Breeding and Cultivation, Shenyang Agricultural University, Shenyang, China; ^3^TieLing Academy of Agricultural Science, Tieling, China

**Keywords:** *CONSTANS-like*, woodland strawberry, flowering, expression profiles, photoperiod

## Abstract

*CONSTANS-like* (*CO-like*) gene is one of the most important regulators in the flowering process of the plant, playing a core role in the photoperiodic flowering induction pathway. In this study, we identified 10 distinct *CO-like* genes (*FveCOs*) in woodland strawberry (*Fragaria vesca*). They were classified into three groups with specific gene structure characteristics or protein domains in each group. The effect of selection pressure on the *FveCOs* in the woodland strawberry was tested by Ka/Ks, and it was shown that the evolution rate of *FveCOs* was controlled by purification selection factors. Intraspecific synteny analysis of woodland strawberry *FveCOs* showed that at least one duplication event existed in the gene family members. Collinearity analysis of woodland strawberry genome with genomes of *Arabidopsis*, rice (*Oryza sativa*), and apple (*Malus* × *domestic*a) showed that *CO-like* genes of *F. vesca* and *Malus* × *domestic*a owned higher similarity for their similar genomes compared with those of other two species. The *FveCOs* showed different tissue-specific expression patterns. Moreover, real-time quantitative PCR results revealed that the expressions of the most *FveCOs* followed a 24-h rhythm oscillation under both long-day (LD) and short-day (SD) conditions. Further expression analysis showed that the individual expression changing profile of *FveCO3* and *FveCO5* was opposite to each other under both LD and SD conditions. Moreover, the expression of *FveCO3* and *FveCO5* was both negatively correlated with the flowering time variation of the woodland strawberry grown under LD and SD conditions, indicating their potential vital roles in the photoperiodic flowering regulation. Further protein interaction network analysis also showed that most of the candidate interaction proteins of FveCO3 and FveCO5 were predicted to be the flowering regulators. Finally, LUC assay indicated that both FveCO3 and FveCO5 could bind to the promoter of *FveFT1*, the key regulator of flowering regulation in the woodland strawberry, and thus activate its expression. Taken together, this study laid a foundation for understanding the exact roles of FveCOs in the reproductive development regulation of the woodland strawberry, especially in the photoperiodic flowering process.

## Introduction

Flowering is a critical growth transition period during growth and development of the plant. To ensure the continuation of species, plants regulate their flowering process accompanying with the external environmental factors such as the temperature and day length. Moreover, plants also adjust their endogenous hormones in response to the external environmental factors to ensure their vegetative and reproductive growth (Song et al., 2013). Flowering regulation is a sophisticated biological process involving various signaling pathways, which have been extensively revealed in the past decades (Perrella et al., 2020). Traditional signaling pathways of flowering regulation are normally known as the photoperiod, vernalization, autonomy, and gibberellin pathway (Mouradov et al., 2002; Hayama and Coupland, 2003; Michaels et al., 2005; Wenkel et al., 2006; Domagalska et al., 2010). The emerging signaling pathways include age pathway, thermosensory pathway, sugar pathway, stress pathway, and hormonal signals to control floral transition (Izawa, 2021). Photoperiodic flowering dominates among these pathways, especially in *Arabidopsis* and rice (Putterill et al., 1995; Yano et al., 2000). According to the day-length requirements in flowering, photoperiodic condition can be described as long-day (LD) condition, short-day (SD) condition, and neutral condition.

CONSTANS (CO) protein is a core transcriptional regulator in the photoperiodic pathway, which is firstly reported in *Arabidopsis* mutant studies. AtCO in *Arabidopsis* accelerated flowering only under long-day (LD) condition via activating the transcriptions of *AtFT* and *AtSOC1* (Valverde, 2011). However, the *CO* homologs can also promote flowering under SD condition in other species. For example, in rice, the *CO* homolog *HEADING DATE 1* (*Hd1*) promoted flowering under SD condition, but suppressed flowering under LD condition, which is completed by regulating the expression of the rice *FT* ortholog *HEADING DATE 3a* (*Hd3a*) (Yano et al., 2000).

The CO homologs were characterized as the important zinc-finger transcription factors (TFs) belonging to a subset of BBX protein family with the specific B-box and CCT domains. The B-box domain located in the N-terminal was found to participate in the protein–protein interaction, while the CCT domain located in the C-terminal owned the nuclear localization function (Putterill et al., 1995; Yano et al., 2000; Robson et al., 2001; Griffiths et al., 2003; Valverde, 2011). The CONSTANS/CONSTANS-like proteins (CO/COLs) can be divided into three major groups according to the divergence of conserved domains (Crocco and Botto, 2013). The CCT domain in the C-terminal can be found in all group members. According to the basis of the consistency of amino acid sequences and the specificity of zinc-binding amino acid residues, the B-box domain can be divided into two types, named as B-box1 (B1) and B-box2 (B2) individually. Group I members owned both B-box1 domain and B-box2 domain, while group II members possessed only B-box1 domain. Group III members owned B-box1 domain and one diverged zinc-finger structure.

Diverse numbers of *CO-like* gene were detected in different plant species, such as 17 members of *CO* family initially identified in *Arabidopsis* (Robson et al., 2001; Crocco and Botto, 2013; Romero-Campero et al., 2013; Wang et al., 2013) and 16 members identified in rice (Griffiths et al., 2003). For horticulture plants, *CO-like* genes have also been widely detected, such as 11 members in *Medicago truncatula* (Wong et al., 2014), 16 members in potato (*Solanum tuberosum*) (Talar et al., 2017), 20 members in grape (*Vitis vinifera*) (Wang et al., 2019), 23 members in tomato (*Solanum lycopersicum*) (Yang et al., 2020), 25 members in banana (*Musa acuminata*) (Chaurasia et al., 2016), and 25 members in Chinese cabbage (*Brassica campestris*) (Song et al., 2015).

The *CO* gene was initially identified as the transcriptional activator of *FLOWERING LOCUS T* (*FT*) using its B-box domain to form a multimeric binding to the *FT* promoter (Samach et al., 2000; Tiwari et al., 2010). FT protein plays the “florigen” role and can directly target the second exon of *LFY*, the master regulator of flower fate, to enhance its expression (Zhu et al., 2020). In *Arabidopsis*, AtCO is considered to be inactive under SD conditions. Flowering time of the *co* mutant is the same under either LD or SD condition. However, cutting down the expression of *AtCO* leads to late flowering under LD condition, while overexpression promotes the flowering process under both LD and SD conditions (Putterill et al., 1995; Robson et al., 2001; Kotake et al., 2003).

In *Arabidopsis*, the expression of *AtFT* was increased by AtCO and AtCOL5 (Hassidim et al., 2009), which were positive regulators of *FT*. However, not all the CO members function as the flowering activator. *AtCOL3* and *AtCOL4* acted as flowering repressors under both LD and SD conditions, while *AtCOL8* and *AtCOL9* delayed flowering only under LD condition (Cheng and Wang, 2005). *Atcol3* mutant could flower earlier under both LD and SD conditions. Further researches demonstrate that AtCOL3 may regulate the expression of *AtFT* by interacting with AtBBX32 to control *Arabidopsis* flowering (Yang et al., 2019).

Functions of CO/COL members were not only restricted in the photoperiodic flowering regulation; remarkable different roles of *CO*/*COL* genes had been reported in various species. For example, in rice, OsBBX5 plays a role in downstream of phytochrome-B receptor, while OsK accelerates the leaf senescence. The ectopic expression of *AtCO* gene in potato can inhibit the potato tuber expansion, while silencing of potato *StCO* gene can promote the potato tuber expansion. In *Glycine max*, *GmCO9* affects root development and is closely related to seed maturation (Huang et al., 2011). *CO* family also mediates various aspects functions of the plant given as follows: *AtCOL3* regulates root growth (Datta et al., 2006), *VviCOL1* plays a major role in bud dormancy (Almada et al., 2009), *CrCO* regulates star synthesis and cell division (Deng et al., 2015), *GmCO9* is closely related to seed competition (Liu et al., 2011), *AtCOL7* regulates branching (Wang et al., 2013), *Ghd2* confers drought sensitivity (Liu et al., 2016), *StCO1* is involved in tuberization (González-Schain et al., 2012), and *MaCOL1* regulates fruit ripening (Chen et al., 2012).

Strawberry (*Fragaria* × *ananassa*), cultivated in different arable regions all over the world, is one of the most important berries characterized by its unique flavor and nutritional value. Fruit quantity and quality directly determine the economic value of strawberry. Early flowering is one of the great advantages of strawberry cultivation with the reduced production time. Compared with other plants, interesting flowering habit is reported in strawberry. For example, while most cultivars of cultivated strawberry are June-bearing SD plants, there are also strawberry cultivars that flower perpetually with no requirement for SD or low-temperature condition, known as the everbearing types (EB). A similar flowering habit also exists in the wild diploid strawberry, whose genome is much simpler than that of the cultivated octoploid strawberry.

At present, studies on strawberry flowering mainly focus on the effects of ambient temperature, photoperiod, or hormone on flowering regulation (Koskela et al., 2012; Sønsteby et al., 2017). Molecular mechanisms research about flowering regulation in strawberry is mainly limited in the function illustration of its FT homolog and TFL1 homolog, which were found function as the florigen and antiflorigen individually (Zhu et al., 2020). Few reports mentioned the possible roles of *CO/COL* genes in woodland strawberry flowering. *FvCO*, a homologous gene of *AtCO* in *F. vesca*, is found to be indispensable for the generation of the bimodal rhythm expression profile of *FvFT1* and thus plays its role in the photoperiodic development of strawberry (Kurokura et al., 2017).

The roles of strawberry *CO/COL* genes in the flowering process have not yet been well elucidated. Hence, in this study, we performed the genome-wide identification of the *CO-like* gene family members in the woodland strawberry based on the high-quality *Fragaria vesca* v4.0.a1 genome database. We provide the detailed molecular information about the *Fragaria vesca CO-like* gene family, including the chromosomal location, sequence homology, introns distribution, motif composition, and evolutionary relationships. The expression of *FveCOs* in different tissues and organs was checked. Meanwhile, their diurnal expression changes in leaf treated with different photoperiodic conditions (LD or SD) were also analyzed. Our results would be valuable for understanding the roles of strawberry *CO-like* genes, especially in the photoperiodic flowering of strawberry.

## Materials and Methods

### Identification of *CO/COL* Genes in Woodland Strawberry

A BLAST search (E-value < 1E^––5^) was performed against woodland strawberry *(F. vesca)* genome data v4.0.a1 in the Genome Database for Rosaceae (GDR^1^) using the full-length amino acid sequences of COs and COLs of *Arabidopsis*. The amino acid sequences of 17 CO/COL proteins in *Arabidopsis* were obtained from NCBI^2^. Then, the sequences of the retrieved woodland strawberry *(F. vesca*) CO/COL candidates (FveCOs) were submitted to the PFAM database^3^ to annotate the unique and conserved protein domains. The amino acid sequences of the B-box and CCT domains in *Arabidopsis* CO/COL proteins peculiar to the members of this family were then used as the query sequences for further confirmation.

The detailed information of identified woodland strawberry *CO-like* genes, including chromosomal location, cDNA length, ORF, and the amino acid (AA), was then downloaded from the GDR. Physicochemical properties of the identified FveCOs proteins, including molecular weight (MV) and isoelectric point (pI), were counted in the ProtParam database^4^. The prediction of subcellular localization was implemented through the PSORT^5^.

### Chromosomal Mapping, Gene Structure, and Multiple Sequence Alignment

Chromosomal positions of *FveCOs* were plotted with MapInspect software^6^.

Gene structure was drawn with Gene Structure Display Server^7^ following the DNA and CDS information of *FveCOs*.

Multiple sequence alignment of FveCOs and other homologs was performed by ClustalW in the MEGA7 software package (Saitou and Nei, 1987; Tamura et al., 2011). Then, the alignment result was illustrated with JalView^8^.

### Phylogenetic Analyzes and Motif Analyzes

Phylogenetic analysis was performed using the CO/COL homologous sequences of several plant species together with the FveCOs. The CO/COL protein sequences used were obtained from the GDR database^9^, Ensembl Plants^10^, NCBI^11^, and Phytozomev10^12^. MEGA7 software was used to construct the phylogenetic tree using the neighbor-joining (NJ) method and Jones–Taylor–Thornton (JTT) model by partial deletion with 2000 bootstrap replications.

The main motifs of FveCOs were characterized by the MEME program^13^. Then, the schematic diagrams of protein domain structure and conserved motif were illustrated with the TBtools software (Chen et al., 2020)^14^.

### Computation of Ka/Ks Values

ParaAT2.0 program^15^ was used to perform the nucleotide sequence alignment of *FveCOs*. Non-synonymous and synonymous substitution rates (denoted as Ka and Ks, respectively) were implemented by Ka/Ks_Calculator program^16^. The ratio of Ka/Ks was used to detect natural selection pressure.

### Collinearity Analysis

Genome data of *F. vesca*, *A. thaliana*, *O. sativa*, and *Malus* × *domestic*a were used to analyze their collinearity and synteny relationships. The genome sequences and genome annotation files were downloaded from Phytozome v10^17^. MCScanX software was used to perform the whole gene collinearity analysis of the three species, and CO-like gene collinearity of the species was also stood out. The chart was manufactured with Circos 0.69, drawing software developed by Perl^18^.

### Analysis of the *cis*-Acting Elements

The upstream sequences (2000 bp) of *FveCOs* were collected for the analysis of cis-acting elements distributed in their promoter regions. The corresponding analysis was performed by online tools PlantCARE (Lescot et al., 2002)^19^, and then, the results were exported with TBtools software^20^.

### Prediction of *FveCOs* Interaction Proteins

The interaction networks of FveCO proteins were predicted and constructed by the STRING v11.0^21^. The active interaction sources include text mining, experiments, databases, co-expression, neighborhood, gene fusion, and co-occurrence. The minimum required interaction score was set as 0.400.

### Expression Detection of *FveCOs*

Tissue-specific expression analyzes and diurnal expression analyzes were carried out in the woodland strawberry LD-flowering accession “Ruegen.” For other analyzes, the plants were field-grown in a greenhouse under natural LD conditions during the spring in ShenYang (Liaoning, China; 41°N, 123°E).

Tissue-specific expression detection was carried out with the samples of root (R), petiole (P), leaf (L), flower (F), shoot apex (SA), green fruit (GF), white fruit (WF), turning red fruit (TF), and red fully fruit (RF). All the samples were frozen in liquid nitrogen and laid at –80°C before total RNA was extracted using a modified cetyltrimethylammonium bromide (CTAB) method as described in Koskela et al. (2012). The full-length cDNAs were synthesized using the PrimeScript RT reagent Kit (TaKaRa).

For diurnal expression analysis, the woodland strawberry “Ruegen” plants with three true leaves were moved to the artificial illumination incubators under 12-h light and 12-h dark conditions for 10 days. Then, the plants were moved into two artificial illumination incubators with different photoperiodic treatments, 25°C/18°C in day/night under LD (16-h light) and SD (8-h light) conditions, respectively. Leaves were then collected to detect the diurnal expression profiles of *FveCOs* at the beginning of the light phase (zeitgeber time 0, ZT0) under different photoperiodic conditions. The leaves were collected as materials every 4 h over 24 h, and the last time point is ZT24.

Pearson’s correlation analyzes were performed with SPSS Statistics 22.0 (IBM Corporation, Armonk, USA) to explore the relationships between the flowering time and relative expression of *FveCO3* and *FveCO5*. Leaves of three plants under each mentioned photoperiod were sampled for expression detection of *FveCO3* and *FveCO5* when the first inflorescence appeared.

qRT-PCR was performed on the CFX96 Real-Time PCR System (Applied Biosystems, Foster City, CA, United States) using the SYBR Premix Ex Taq Kit (TaKaRa) according to the manufacturer’s protocol. The *FveActin* served as an internal control. The relative expression of genes was presented by the 2^–ΔΔ*Ct*^ method. All of the above samples were executed independently in triplicate. Primers used in this study are listed in Supplementary Table 1.

### Dual-Luciferase Assays

The dual-luciferase reporter assay was carried out. The 35S:FveCO3 and 35S:FveCO5 vectors were constructed (Supplementary Figure 1) and used as effectors, and the 2-Kb *FveFT1* promoter was inserted into the pGreen II 0800 vector and used as the reporter. The constructed vectors were transformed into *Agrobacterium* strain GV1301. *Agrobacterium* strains were introduced into tobacco (*Nicotiana tabacum*) leaves. The luciferase fluorescence and luciferase signal intensity were imaged and measured after three days using a living fluorescence imager (Lb985, Berthold, Germany).

## Results

### Identification of *CO-like* Genes in Woodland Strawberry

To survey the *CO*-like members in the woodland strawberry, a genome-wide search against the GDR database was performed via selecting the typical B-box and CCT domains. A total of 10 distinct genes were identified as putative members of the woodland strawberry *CO*-*like* members. The gene names were entitled according to the order in which they were found. The detailed information of these genes is shown in Table 1.

**TABLE 1:** Sequence analysis of *FveCOs*.

Gene names	Gene ID	Length of cDNA (bp)	Length of ORF (bp)	AA	Chromosome	Position	MW (kDa)	pI	Prediction of protein location
*FveCO1*	gene00355	2376	1437	478	LG7	90927..92118	51.77	5.66	Nuclear
*FveCO2*	gene03742	1473	1353	450	LG4	962863..964335	50.33	5.56	Nuclear
*FveCO3*	gene04172	1863	1158	385	LG6	31553425..31557231	42.35	5.35	Nuclear
*FveCO4*	gene14981	1020	939	312	LG2	35460923..35461942	34.5	6.33	Nuclear
*FveCO5*	gene15552	2368	1254	417	LG6	23430145..23432512	45.25	5.45	Nuclear
*FveCO6*	gene24941	1664	1182	393	LG2	562983..564196	43.58	5.74	Nuclear
*FveCO7*	gene25171	1726	1374	456	LG5	26696242..26697967	51.23	5.56	Nuclear
*FveCO8*	gene27383	1191	942	356	LG4	10584825..10586015	38.99	5.63	Nuclear
*FveCO9*	gene03650	1269	912	303	LG4	24743480..24758335	33.28	6.25	Nuclear
*FveCO10*	gene03651	2554	1416	471	LG4	24759237..24764662	52.96	5.62	Nuclear

The cDNA length of the *FveCOs* ranged from 1020 bp to 2376 bp, following the polypeptide sequences varying from 312 aa to 478 aa and the molecular weights of 51.77 kDa to 34.5 kDa. The prediction of FveCOs proteins showed that they were all located to the cell nucleus (Table 1).

**FIGURE 1:**
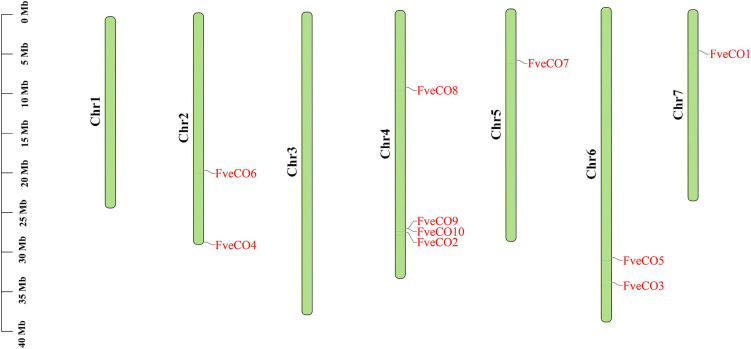
Chromosome localization map of *FveCOs*. The light green vertical bars with various lengths represent woodland strawberry chromosomes. Short black vertical lines indicate the position of each *FveCOs.*

Chromosomal distribution detection of *FveCOs* determined by the chromosome mapping indicated that these genes were unevenly distributed on five chromosomes (Figure 1). In detail, *FveCO4* and *FveCO6* were both located on chromosome 2, *FveCO1* and *FveCO7* were independently located on chromosomes 5 and 7, *FveCO2*, *FveCO8*, *FveCO9*, and *FveCO10* were located on chromosome 4, and *FveCO3* and *FveCO5* were located on chromosome 6.

### Phylogeny and Multiple Sequence Alignment Analyzes of *FveCOs*

**FIGURE 2:**
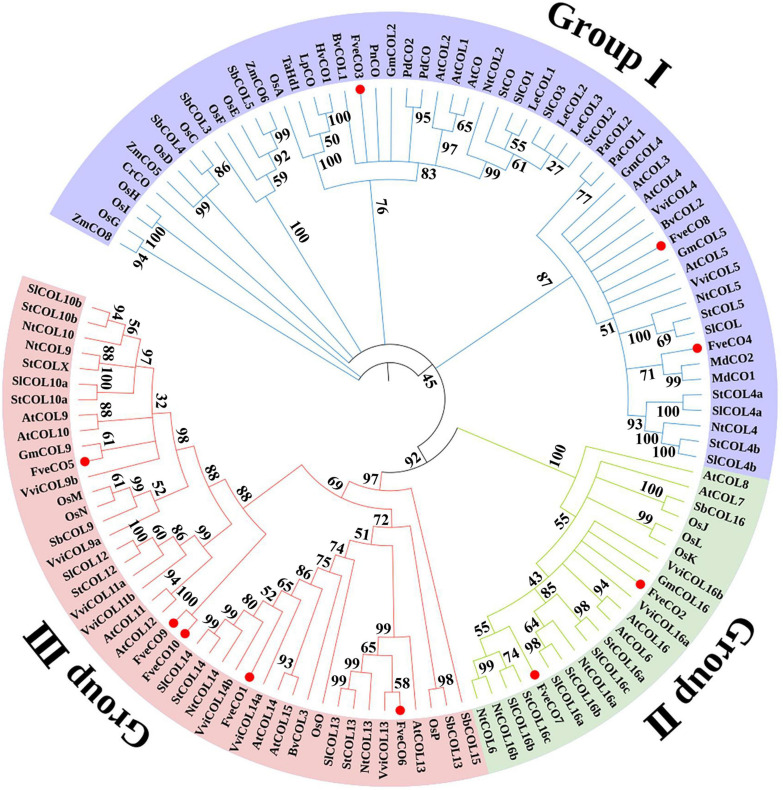
Phylogenetic analysis of CONSTANS/CONSTANS-like (CO/COL) homologs in different species. The clades of groups I, II, and III are marked in green, blue, and pink, respectively. *Fragaria vesca constans-like genes* (FveCOs) are indicated by red points. At, *Arabidopsis thaliana*; Ca, *Capsicum annuum*; Gm, *Glycine max*; Hv, *Hordeum vulgare*; Lt, *Lolium temulentum*; Md, *Malus domestica*; Nt, *Nicotiana tabacum*; Os, *Oryza sativa*; Ph, *Petunia hybrida*; Sb, *Sorghum bicolor*; Sl, *Solanum lycopersicum*; St, *Solanum tuberosum*; Ta, *Triticum aestivum*; Vv, *Vitis vinifera*; Zm, *Zea mays*.

To fully identify the evolutionary relationship of CO homologs belonging to the woodland strawberry and other plant species, a phylogenetic tree was constructed with 121 CO-like amino acid sequences of 20 plant species, including 17 of *Arabidopsis*, 13 of tomato, 10 of tobacco, 16 of rice, 2 of apple, and so on. The results (Figure 2) suggested that these CO-like proteins could be subdivided into three groups, which are consistent with the previous findings. As shown in the tree, three *Fragaria vesca* CO homologs, namely, FveCO3, FveCO4, and FveCO8, belong to group I, two homologs, namely, FveCO2 and FveCO7, belong to group II, while the other five homologs, namely, FveCO1, FveCO5, FveCO6, FveCO9, and FveCO10, belong to group III.

Protein structures and evolutionary relationships could be elucidated by multiple sequence alignment. The alignment results of the amino acid sequences of FveCOs showed that all 10 FveCOs contained at least one B-box domain and one CCT domain. The identity of 10 FveCOs amino acid sequences ranged from 15.4% to 49.9% (Supplementary Figure 2).

According to the consistency difference of the amino acid sequence of B-box domain and the specificity of zinc ion-binding amino acid residues, the B-box domain can be further divided into two types: B-box1 (B1) and B-box2 (B2). Besides the B1 and B2 domains, an additional diverged zinc-finger structure (DZF) was also found in some CO-like proteins of *Fragaria vesca*.

B-box1 domain is the most conserved region in all the FveCOs proteins and is composed of approximately 40 residues, which owned the consensus C-X2-C-X7-9-C-X2-D-X4-C-X2-C-X3-4-H-X4-8-H, where X can be any amino acid. CCT domains are also highly conserved in FveCOs containing 43–45 amino acid residues.

**FIGURE 3:**
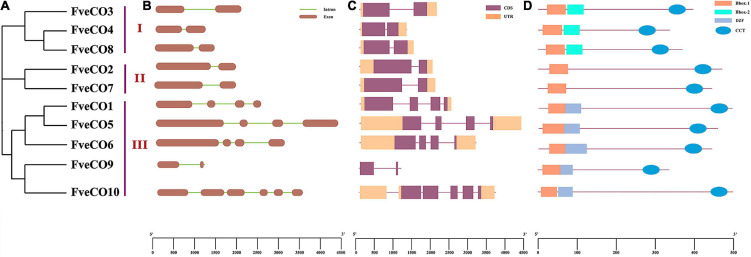
Genomic and protein structures of *FveCOs*. **(A)** Phylogenetic analysis of 10 *FveCO* genes which are categorized them into three groups. **(B)** The intron distribution. The exons and introns are represented by dark ochre boxes and light green lines, respectively. **(C)** The CDS and UTR are represented by purple boxes and orange boxes, respectively. **(D)** The domain distribution of FveCOs. The CCT, B-box1, B-box2, and DZF are represented by light blue ovals, orange boxes, light green boxes, and blue–gray boxes, respectively.

Thus, according to the domain combination, FveCOs can be grouped into three types, similar to the three clades shown in the phylogenetic tree (Figure 3D). Members in type I (FveCO3, FveCO4, and FveCO8) own two B-box domains and one CCT domain (B1 + B2 + CCT), members in type II (FveCO2 and FveCO7) own only one B-box domain (B1) and one CCT domain (B1 + CCT), while for members in type III (FveCO1, FveCO5, FveCO6, FveCO9, and FveCO10), besides a typical B1-type B-box domain and one CCT domain, the diverged zinc-finger structure domain was also detected (B1 + DZF + CCT).

Meme online software was used to further analyze the amino acid sequence similarity of those domains. The B-box domain is rich in amino acids such as A, C, D, and L, while the CCT domain is rich in arginine (R) and lysine (K) (Supplementary Figure 3). Although the B1, B2, and the DZF domains are all belonging to the zinc-finger domain, their amino acid sequences are variant, even in B1 and B2 (Supplementary Figure 3).

### Gene Structure Analysis of *FveCOs*

Intron distribution is important for the gene selective splicing, allowing a gene to produce different proteins. The distribution of the *FveCO*s introns was identified by the comparative analyzes of *FveCOs* DNA sequences with their coding sequences. Two exons and one intron were detected in all the members belonging to group I (*FveCO3, FveCO4*, and *FveCO8*) and group II (*FveCO2* and *FveCO7*). For members in group III, most of them own four exons and three introns, except *FveCO9* and *FveCO10*. *FveCO9* has only one intron just like members in groups I and II, while five introns were detected in *FveCO10* (Figure 3B).

The introns of *FveCO*s are 81 bp–1114 bp in length separately, leading to a large variation in their genomic length. The CDS and UTR information of *FveCOs* is listed in Figure 3C.

### Synteny Analysis of *FveCOs*

**FIGURE 4:**
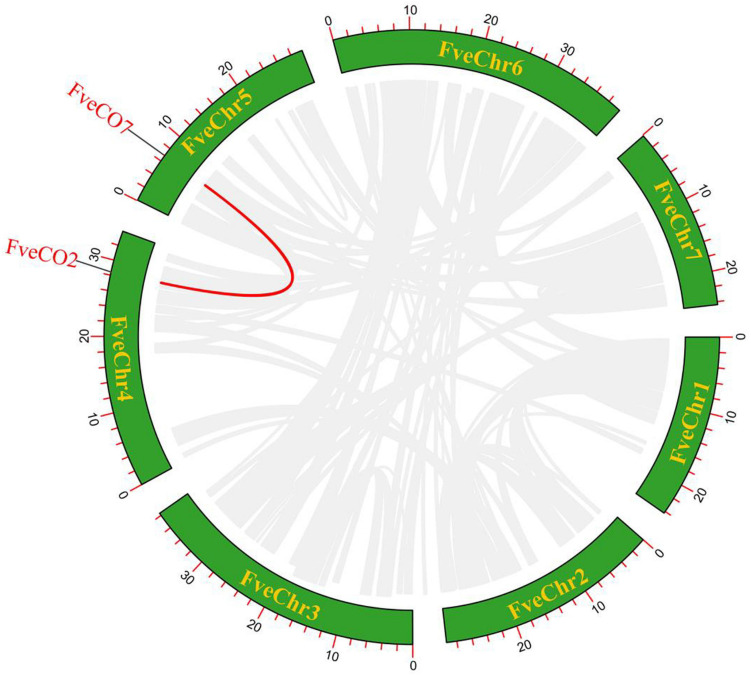
Schematic representation of the interchromosomal relationships between *FveCOs* in the *Fragaria vesca* genome. Red line indicates the colinear gene pair, and gray lines indicate the syntenic blocks.

To estimate the evolutionary character of woodland strawberry *FveCO* genes, the replication events about this gene family in the intraspecific and interspecific genomes were also analyzed. The results implied that only one pair of duplicate genes (*FveCO2*/*FveCO7*) was found in the genome of the woodland strawberry, which may be the result of tandem replication or whole-genome replication (WGD) (Figure 4).

To further explore the selection pressure between *FveCO* duplicate genes, we calculated the Ka, Ks, and Ka/Ks values of paralogous genes (Table 2). The divergence time of the woodland strawberry was estimated as 9.4 Mya (million years ago). Moreover, with the Ks value, we also calculate the substitutions rate of per site per year as 5.8 × e^–8^.

**TABLE 2:** Ka/Ks analysis for the duplicated *FveCO* paralogs.

	Ka	Ks	Ka/Ks	Purifying selection	Duplicate type
*FveCO2/FveCO7*	0.4225	2.9676	0.1423	Yes	Segmental

**FIGURE 5:**
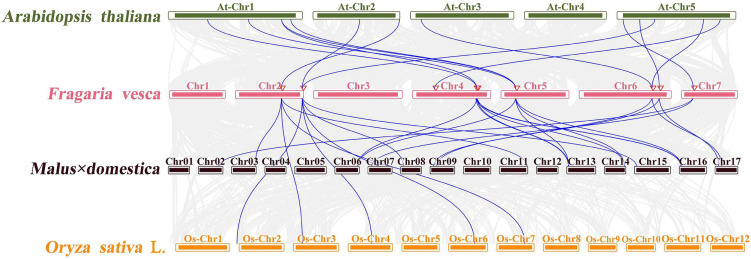
Microsynteny of CONSTANS-like *(COL)* regions across *Fragaria vesca*, *M* × *domestica*, *O. sativa*, and *A. thaliana*. The *F. vesca*, *M* × *domestica*, *O. sativa*, and *A. thaliana* chromosomes are shown in different colors. The number on each chromosome box indicates the number of chromosomes. Blue lines represent the syntenic relationships between *AtCOLs*, *MdCOs*, *OsCOLs*, and *FveCOs* regions. Light gray lines represent the syntenic relationships between genome wide of *Arabidopsis*, woodland strawberry, apple, and rice.

Moreover, to explore the evolution mechanism and biochemical features of the *FveCOs*, a collinearity comparison of *Fragaria vesca* genome with genomes of *Arabidopsis*, rice, and apple, belonging to dicotyledon plant, monocotyledon plant, and Rosaceae plant, respectively, was also performed. The results showed that there are 12 collinear gene pairs between *F. vesca* and *A. thaliana*, 17 pairs between *M* × *domestica* and *F. vesca*, and 5 pairs between *O. sativa* and *F. vesca* (Figure 5). Orthologous gene numbers identified between *F. vesca* and *M* × *domestica*, which are all belonging to Rosaceae, were much larger than those identified between woodland strawberry and *Arabidopsis* and rice.

Both *FveCO4* and *FveCO6* were located on the second chromosome, and their orthologous gene pairs were detected in the rice genome. Orthologous gene pairs of *FveCO1*, *FveCO2*, *FveCO3*, *FveCO4*, *FveCO5*, *FveCO6*, *FveCO7*, and *FveCO8* were identified in both apple and *Arabidopsis*, and the remaining *FveCOs* were not present in any of the duplicated blocks. These results suggested that those genes are highly conservative and the collinear gene pairs may come from the common ancestor before evolution.

### *Cis*-Element Analysis of Woodland Strawberry *FveCOs*

Promoters with 2 kb in length of each *FveCO* members were used to identify the putative cis-acting regulatory elements (CREs) with the PlantCARE database. Totally, 43 kinds of CREs were identified (Supplementary Table 2). Among them, there are 283 core promoter elements (TATA box) and 109 common *cis*-acting elements (CAAT box).

The other *cis*-acting elements identified might be divided into three main kinds, namely hormone response element, light response element, and stress response element.

**FIGURE 6:**
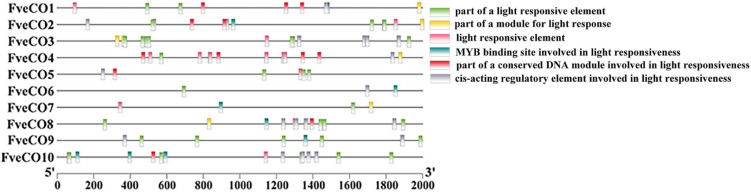
Distribution of the major light-related *cis*-elements in the promoter sequences of 10 *FveCO* genes. Different *cis*-elements are represented by different symbols as indicated.

*Fragaria vesca constans-like genes* may also play crucial roles in the regulation of photoperiod flowering just like their reported homologs; thus, we further analyzed the light response elements distributed in their promoters. As shown in Figure 6, a large number of light-responsive elements were detected in all the promoter regions of 10 *Fve*CO genes, while the kinds and amounts are various. Promoter of *FveCO3* owns 21 light-responsive *cis*-elements with the maximum quantity, while promoter of *FveCO6* only has three light-responsive *cis*-element with the minimum number.

### Expression of *FveCOs* in Different Tissues

**FIGURE 7:**
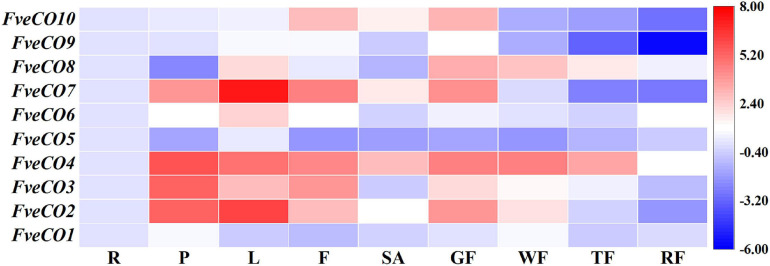
Expression of *FveCOs* in different woodland strawberry tissues. Different colors of heat map represent the expression levels. The tissues of the samples are as follows: R, root; P, petiole; L, leaf; F, flower; SA, shoot apex; GF, green fruit; WF, white fruit; TF, turning red fruit; RF, red fully fruit. For each gene, the expression level was set to 1 in the root, and the corresponding fold changes were calculated in other tissues. The gene expression heatmap was generated on the log base 2 average expression fold values.

The qRT-PCR results showed that *FveCO*s were variously expressed in different tissues (Figure 7). All *FveCO* genes exhibited higher expression levels in leaves and petioles than those in other tissues. For fruits in different development stages, *FveCOs* are mainly expressed in green fruit, especially for the expression of *FveCO2*, *FveCO4*, *FveCO7*, *FveCO8*, and *FveCO10*. It was also found that the lowest *FveCO* expression level was detected in the fully red fruit.

Among all of these genes, *FveCO3* and *FveCO4* showed similar high expression levels in different tissues, while the expression of *FveCO9* was border on the minimum in each tested tissue. The specific and varied expression profiles of *FveCO*s in different tissues suggest that they may play diverse roles.

### Expression Profile of *FveCOs* in Photoperiodic Flowering

According to the previous studies, the expression of the *CO-like* genes exhibits a circadian rhythm profile in most plant species (Suárez-López et al., 2001; Wang et al., 2013; Fu et al., 2015; Chaurasia et al., 2016). Therefore, to evaluate the potential functions of *FveCOs* in photoperiodic flowering, we detected their diurnal expression profiles over a 24-h period at 4-h intervals under LD and SD conditions, separately.

**FIGURE 8:**
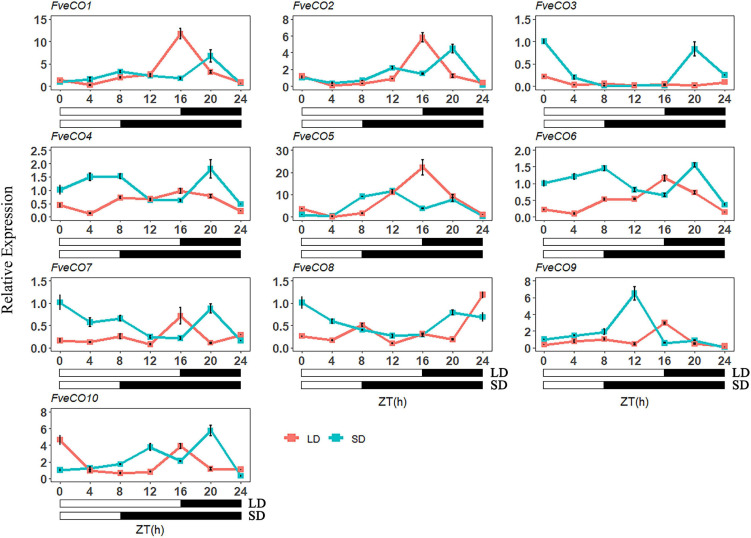
Diurnal expression pattern of *FveCOs* under long-day (LD) and short-day (SD) conditions. The collection of samples was started at the beginning of the light phase (zeitgeber time 0, ZT0) and continued every 4 h over 24 h under LD (16-h light/8-h dark) and SD (8-h light/16-h dark) conditions. Each value is the mean ± SD of three biological replicates.

As shown in Figure 8, three genes, including *FveCO1*, *FveCO2*, and *FveCO5*, owned similar expression patterns under both photoperiodic conditions, with the highest expression level that appeared at ZT16 h under LD condition and then gradually reduced at night. However, the expression levels of these three genes slightly increased during the day of SD and peaked at midnight. In addition, the peak expressions of these genes were higher in plants grown under LD condition than under SD condition.

*FveCO4*, together with *FveCO6, FveCO7*, and *FveCO10*, showed the consistent expression pattern under SD and LD conditions, which were slightly similar to that of *FveCO1*, *FveCO2*, and *FveCO5.* The expression of *FveCO4*, *FveCO6*, *FveCO7*, and *FveCO10* under SD condition was detected higher separately than that under LD condition. The expression peaks appeared at ZT20 h and ZT16 h for SD and LD conditions, respectively. Under SD condition, their expression decreased rapidly to the lowest level.

### Correlation of Woodland Strawberry Flowering Time and the *FveCOs* Expression

**FIGURE 9:**
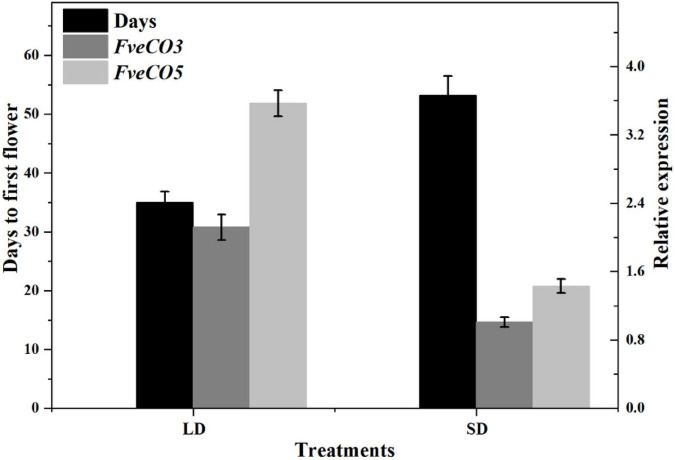
Negative correlation of *FveCO3* and *FveCO5* expression with flowering time in woodland strawberry “Ruegen.” The black bars indicate the days needed to flower (flowering time); the gray bar and light gray bar indicate the expression of *FveCO3* and *FveCO5*, respectively. Ten plantlets were used for each. Three independent samples were used in the expression analysis. Values are the mean ± SD.

To further explore the potential function of *FveCOs* on strawberry flowering time, we also investigated the correlation between the flowering time and the expression levels of *FveCOs.* In all the *FveCOs*, only the expression of *FveCO3* and *FveCO5* showed the correlation with the flowering time. The results showed that the expression levels of *FveCO3* and *FveCO5* were all negatively correlated with the flowering time under both LD and SD conditions (*r* = –0.949, –0.964, –0.936, and –0.891, respectively) (Figure 9). Plants under LD condition showed earlier flowering time with higher expression levels of both *FveCO3* and *FveCO5*, while plants grown under SD condition owned the lower expression levels of both *FveCO3* and *FveCO5*.

### *FveCO3* and *FveCO5* Activate the Expression of *FveFT1*

**FIGURE 10:**
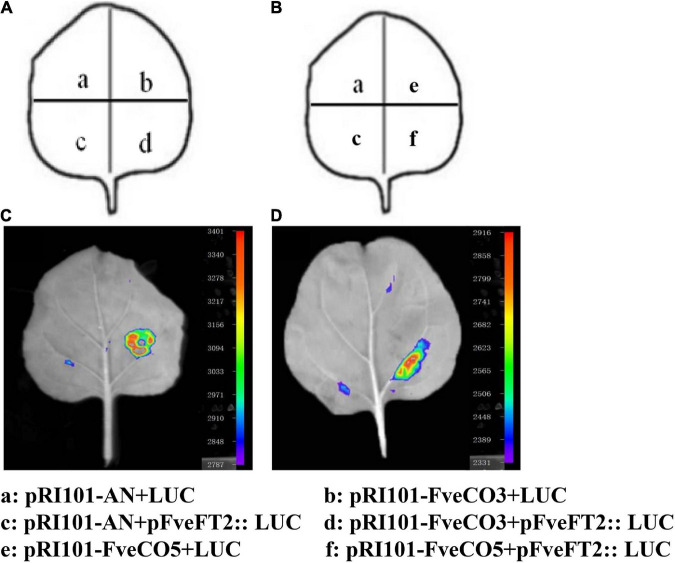
*FveCO3* and *FveCO5* regulate *FveFT1* expression by binding to its promoter. **(A,B)** Schematic diagram of the reporter and effectors used in luciferase reporter assay. Luciferase reporter assay showing that FveCO3 and FveCO5 regulate *FveFT1* expression **(C,D)**, respectively. The FveCO3 and FveCO5 effector and the *proFveFT1* reporter were coinfiltrated into tobacco leaves, and the luciferase signal was measured.

To explore how *FveCO3* and *FveCO5* regulate photoperiodic flowering in the woodland strawberry, luciferase reporter assay was carried out. The promoter sequence of 2000-bp upstream of the ATG codon of *FveFT1* was cloned and inserted into the upstream of LUC reporter gene. The effector plasmid containing 35S-FveCO3 and 35S-FveCO5 construct was co-transfected into tobacco leaves, respectively. The luciferase assays indicated that co-expression of 35S-FveCO3 and *proFveFT1-LUC* or 35S-FveCO5 and *proFveFT1-LUC* resulted in much stronger luminescence signals than any other points (Figure 10). These results showed that FveCO3 and FveCO5 could bind to the *FveFT1* promoter individually and thus activate the transcription of the corresponding gene.

**FIGURE 11:**
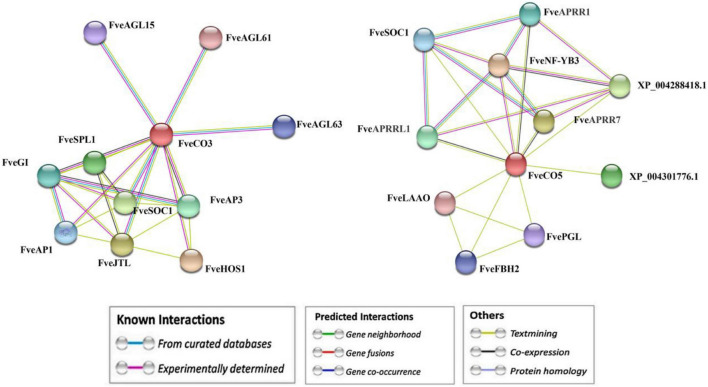
Interaction network prediction of *FveCO3* and *FveCO5* with other proteins. The *Fragaria vesca constans-like genes* (*FveCOs*) are marked in red.

Moreover, the interaction networks of *FveCO3* and *FveCO5* with other flowering-related proteins were predicted and constructed by the STRING v11.0 (see the footnote 21). As shown in Figure 11, multiple genes were screened as the candidate interactors of FveCO3 or FveCO5, including FveSOC1, FveHOS1, FveAGLs, FveGI, FveFBH, and FveAP1.

## Discussion

### Molecular Characteristics of *FveCOs* in Woodland Strawberry

Normally, plants can be classified as either SD or LD type according to their flowering response to the photoperiod. In strawberry, two types of flowering habit exist in different varieties, known as the June-bearing SD type and perpetually flowering everbearing type. For the conserved function of CO-FT module in plant photoperiodic flowering regulation, the characterization of the roles of *CO-like* genes in strawberry flowering habit variation is important for its breeding.

Actually, *CO-like* genes of strawberry had been reported in some previous studies. Kurokura reported one woodland strawberry *CO* homolog (gene 04172) and another nine putative CO-like proteins in *F. vesca* genome (V1.1) using the B-box domain as the search query (Kurokura et al., 2017). Among those putative CO-like proteins, six proteins are included in the FveCOs we reported here, while the CCT domain cannot be detected in the remaining four proteins they reported, so those four proteins should not be classified as CO-like proteins.

Using the updated *Fragaria vesca* genome database v4.0.a1, here we reported the 10 non-redundant FveCO candidates, including the new four ones which had not been reported. All the FveCO candidates contain both B-box domain and CCT domain (Figure 3). The 10 identified FveCOs can be divided into three groups depending on the phylogenetic analysis (Figures 2, 3), which was consistent with the reports in other plants (Putterill et al., 1995; Cheng and Wang, 2005; Hassidim et al., 2009; Song et al., 2015).

Although the *Arabidopsis* genome size (130 Mb) is much smaller than that of the woodland strawberry (230 Mb), the number of *Arabidopsis COL* member is much larger than that in the woodland strawberry (17:10). The gene structure and conserved domain of FveCOs are similar to the homologs of other species. Conserved domain-based CO proteins can also be divided into three subfamilies in the woodland strawberry. However, the gene structure classification of these FveCOs proteins is quite complex. In group III, the gene structures of *FveCO9* and *FveCO10* were different, but their amino acid sequences encoded were similar to each other. Such variation may come from the duplication, variation and recombination of exons, or the insertion and loss of introns in the process of genome evolution.

The existing of duplicated genes implied that independent duplication events existed besides the whole-genome duplication event. Only one pair of *FveCOs* (*FveCO2/7*) is identified to be involved in fragment replication. It should be the reason that the woodland strawberry genome experienced few replication events as a kind of diploid variety. Segmental duplication was a kind of genome replication. Tandem duplications were characterized as multiple members of one family occurring within the same intergenic region or in neighboring intergenic regions. The most representative tandem replication genes are adjacent homologous genes on a single chromosome (Moore and Purugganan, 2003; Yu et al., 2005). In this study, *FveCO9* and *FveCO10* are adjacent genes on chromosome 4. Therefore, FveCO9 and FveCO10 are probably the results of tandem replication.

Collinearity analysis between the genomes of the woodland strawberry and other three plants suggested that the *FveCOs* own the highest relationship with their homologs in apple, followed by *Arabidopsis thaliana*, and the lowest relationship with rice orthologous gene. Such results are consistent with their taxonomical relationship: Strawberry and apple are both Rosaceae plants.

The expansion of all *CO* genes occurs with the divergence of the plants of the same family. The subclasses of *CO* genes extend from the common ancestral genes in a species-specific manner, which existed before the diversification of the same family lineage.

### Expression Profile of *FveCOs* Indicates Their Potential Functions in the Photoperiodic Flowering Regulation

Most of the *FveCO* genes are preferentially expressed in leaf and petiole (Figure 7), which are consistent with the findings in other species (Almada et al., 2009; Tan et al., 2016; Wu et al., 2017). As leaf is the organ in which the plant percepts the light, such result strongly demonstrated that *FveCOs* might also involve in the photoperiodic sensitive response. Besides the leaf and petiole, the expression of *FveCOs* could also be detected in many tissues except the root and red fully fruit, which implies that *FveCO* members might also function in other multiple developmental aspects of the woodland strawberry. On the contrary, the expression of the different *FveCO* members could be detected in the same tissue, which might result in their functional redundancy.

At the same time, our result also showed that the expression of most *FveCO* genes can be regulated by circadian clock (Figure 8), which resulted in their diurnal expression changes. The one-day periodic changing expression profile is the prominent character of almost all the CO-like genes (Martin et al., 2004; Pan et al., 2021). When treated with different photoperiods, the *FveCOs* showed different diurnal expression patterns under SD or LD condition. *FveCO* genes showed different circadian rhythms, including at least four peaks at different ZT time points. Among them, the expression of *FveCO1*, *FveCO2*, *FveCO5*, and *FveCO9* showed notable expressional amplitudes during the circadian rhythms at the time points of measurement in 24 h under both LD and SD conditions.

Interestingly, it was found that the individual diurnal expression profile of *FveCO3* and *FveCO5* was opposite to each other under both LD and SD conditions. Further work should be performed to clarify the mechanism about such different expression profiles, such as the transcriptional or posttranscriptional regulation research of the upstream regulators on *FveCO3* and *FveCO5.*

### *FveCO3* and *FveCO5* Function as Potential Flowering Promoters in Woodland Strawberry

Our results showed that, under either LD or SD condition, the expression levels of *FveCO3* and *FveCO5* were both negatively correlated with flowering time (Figure 9). *FveCO3* is the woodland strawberry homolog of *AtCO* belonging to group I type, while *FveCO5* is the homolog of *AtCOL9* which negatively regulates the expression of *AtCO* and *AtCOL9* to inhibit the flowering in LD. Previous studies have shown that the expression of *AtCO* is induced by SD rather than by LD (Putterill et al., 1995; Robson et al., 2001; Kotake et al., 2003; Jang et al., 2008), while the expression of *FveCO3* in SD or LD was similar to each other, and the same expression profile was also detected in *FveCO5*. Further work about the protein interaction networks prediction of FveCO3 and FveCO5 showed that they could interact with many other reported flowering regulators, such as FveSOC1, FveHOS1, FveAGLs, FveGI, FveFBH, and FveAP1(Figure 11). In *Arabidopsis*, SOC1 is one of the direct targets of CO and directly regulates the flowering process (Samach et al., 2000). GI and FBH can both bind to the upstream CO negative regulatory transcription factor CDFs and thus to degrade the CDFs and positively regulates the stability of CO (Toledo-Ortiz et al., 2003; Fornara et al., 2009). GI also promotes the FT expression to regulate flowering (Imaizumi et al., 2005; Sawa and Kay, 2011; Ito et al., 2012; Fornara et al., 2015). Such finding implied that *FveCO3* and *FveCO5* should both function in the woodland strawberry flowering and the mechanism should be more complex than that of *Arabidopsis*. The function of CO/COL proteins mainly depends on their regulation of the expression of the *FT*-like genes, the master regulators in plant flowering process. *RcCO* is the homolog of *FveCO3* in rose, and it regulates the photoperiodic flowering under long-day condition (Lu et al., 2020). RcCO could bind to the CORE motif of the *RcFT* promoter so as to enhance the *RcFT* expression. In the woodland strawberry, *FveFT1* has been identified as the key gene to determine the flowering time (Koskela et al., 2012). In this study, luciferase reporter assay suggested that both FveCO3 and FveCO5 could directly bind to the *FveFT1* promoter individually and thus may promote the flowering process by transcriptional regulation. Similar upregulation of *FveFT1* was also reported in the strawberry *FveCO* overexpression lines (Kurokura et al., 2017). Those findings suggested that the CO-FT module also functions in the photoperiodic flowering of strawberry.

## Conclusion

Totally, 10 distinct *CO*-like genes (*FveCOs*) in the woodland strawberry (*F. vesca*) were identified. The expression analysis indicated that multiple *FveCO* genes were highly responsive to the photoperiodic induction. Both *FveCO3* and *FveCO5* are potential positive regulators for photoperiodic flowering, which is different from their individual homologs in *Arabidopsis*. FveCO3 and FveCO5 can bind to the promoter of *FveFT1*, the key reported flowering regulator. The mechanism of *FveCO3* and *FveCO5* in strawberry flowering regulation should be deeply clarified with further work.

## Brief Summary

Ten distinct *CO-like* genes (*FveCOs*) were identified in the woodland strawberry (*F. vesca*). The expression of *FveCO3* and *FveCO5* was both negatively correlated with the flowering time variation of the woodland strawberry grown under both long-day and short-day conditions. FveCO3 and FveCO5 may function in flowering induction *via* the photoperiodic regulation in the woodland strawberry.

## Data Availability Statement

The original contributions presented in the study are included in the article/Supplementary Material, further inquiries can be directed to the corresponding author.

## Author Contributions

YL designed the research. XZ, FY, QG, and YW performed the experiments. XZ and YL analyzed the data. XZ wrote the manuscript. YL and ZZ assisted with interpretation of results, manuscript writing, and revision. All authors read and approved the manuscript.

## Conflict of Interest

The authors declare that the research was conducted in the absence of any commercial or financial relationships that could be construed as a potential conflict of interest.

## Publisher’s Note

All claims expressed in this article are solely those of the authors and do not necessarily represent those of their affiliated organizations, or those of the publisher, the editors and the reviewers. Any product that may be evaluated in this article, or claim that may be made by its manufacturer, is not guaranteed or endorsed by the publisher.

## Footnotes

1
https://www.rosaceae.org/

2
https://www.ncbi.nlm.nih.gov/

3
http://pfam.xfam.org/

4
https://www.expasy.org/

5
https://www.genscript.com/psort.html

6
http://mapinspect.software.informer.com/

7
http://gsds.cbi.pku.edu.cn/

8
https://www.jalview.org/

9
https://www.rosaceae.org/

10
https://plants.ensembl.org/index.html

11
http://www.ncbi.nlm.nih.gov

12
https://phytozome-next.jgi.doe.gov/

13 http://meme-suite.org/tools/meme, v5.3.3

14
https://github.com/twdb/tbtools

15
https://ngdc.cncb.ac.cn/tools/paraat

16
https://ngdc.cncb.ac.cn/tools/kaks

17
https://phytozome-next.jgi.doe.gov/

18
http://www.circos.ca/software/download/circos/

19
http://bioinformatics.psb.ugent.be/webtools/plantcare/html/

20
https://github.com/twdb/tbtools

21
https://string-db.org/

## References

[B1] Almada R. Cabrera N. Casaretto J. A. Ruiz-Lara S. Villanueva E. G. (2009). VvCO and VvCOL1, two CONSTANS homologous genes, are regulated during flower induction and dormancy in grapevine buds. *Plant Cell Rep.* 28 1193–1203. 10.1007/s00299-009-0720-4 19495771

[B2] Chaurasia A. K. Patil H. B. Azeez A. Subramaniam V. R. Krishna B. Sane A. P. (2016). Molecular characterization of CONSTANS-Like (COL) genes in banana (*Musa acuminata* L. AAA Group, cv. Grand Nain). *Physiol. Mol. Biol. Plants.* 22 1–15. 10.1007/s12298-016-0345-3 27186015 PMC4840155

[B3] Chen C. J. Chen H. Zhang Y. Thomas H. R. Frank M. H. He Y. (2020). TBtools: an integrative toolkit developed for interactive analyses of big biological data. *Mol. Plant.* 13 1194–1202. 10.1016/j.molp.2020.06.009 32585190

[B4] Chen J. Chen J. Y. Wang J. N. Kuang J. F. Shan W. Lu W. J. (2012). Molecular characterization and expression profiles of MaCOL1, a CONSTANS-like gene in banana fruit. *Gene* 496 110–117. 10.1016/j.gene.2012.01.008 22285923

[B5] Cheng X. F. Wang Z. Y. (2005). Overexpression of COL9, a CONSTANS-LIKE gene, delays flowering by reducing expression of CO and FT in *Arabidopsis thaliana*. *Plant J.* 43 758–768. 16115071 10.1111/j.1365-313X.2005.02491.x

[B6] Crocco C. D. Botto J. F. (2013). BBX proteins in green plants: insights into their evolution, structure, feature and functional diversification. *Gene* 531 44–52. 10.1016/j.gene.2013.08.037 23988504

[B7] Datta S. Hettiarachchi G. H. Deng X. W. Holm M. (2006). *Arabidopsis* CONSTANS-LIKE3 is a positive regulator of red light signaling and root growth. *Plant Cell* 18 70–84. 10.1105/tpc.105.038182 16339850 PMC1323485

[B8] Deng X. D. Fan X. Z. Li P. Fei X. W. (2015). A photoperiod-regulating gene CONSTANS is correlated to lipid biosynthesis in *Chlamydomonas reinhardtii*. *Biomed Res. Int.* 2015:715020. 10.1155/2015/715020 25654119 PMC4310486

[B9] Domagalska M. A. Elzbieta S. Ferenc N. Davis S. J. (2010). Genetic analyses of interactions among gibberellin, abscisic acid, and brassinosteroids in the control of flowering time in *Arabidopsis thaliana*. *PLoS One* 5:e14012. 10.1371/journal.pone.0014012 21103336 PMC2984439

[B10] Fornara F. de Montaigu A. Sánchez-Villarreal A. Takahashi Y. Ver Loren. Van. Themaat E. Huettel B. (2015). The GI-CDF module of *Arabidopsis* affects freezing tolerance and growth as well as flowering. *Plant J.* 81 695–706. 10.1111/tpj.12759 25600594 PMC5006856

[B11] Fornara F. Panigrahi K. C. Gissot L. Sauerbrunn N. Rühl M. Jarillo J. A. (2009). *Arabidopsis* DOF transcription factors act redundantly to reduce CONSTANS expression and are essential for a photoperiodic flowering response. *Dev. Cell* 17 75–86. 10.1016/j.devcel.2009.06.015 19619493

[B12] Fu J. X. Yang L. W. Dai S. L. (2015). Identification and characterization of the CONSTANS-like gene family in the short-day plant *Chrysanthemum lavandulifolium*. *Mol. Genet. Genomics* 290 1039–1054. 10.1007/s00438-014-0977-3 25523304

[B13] González-Schain N. D. Díaz-Mendoza M. Zurczak M. Suárez-López P. (2012). Potato CONSTANS is involved in photoperiodic tuberization in a graft-transmissible manner. *Plant J.* 70 678–690. 10.1111/j.1365-313X.2012.04909.x 22260207

[B14] Griffiths S. Dunford R. P. Coupland G. Laurie D. A. (2003). The evolution of CONSTANS-like gene families in barley, rice, and *Arabidopsis*. *Plant Physiol.* 131 1855–1867. 10.1104/pp.102.016188 12692345 PMC166942

[B15] Hassidim M. Harir Y. Yakir E. Kron I. Green R. M. (2009). Over-expression of CONSTANS-LIKE 5 can induce flowering in short-day grown *Arabidopsis*. *Planta* 230 481–491. 10.1007/s00425-009-0958-7 19504268

[B16] Hayama R. Coupland G. (2003). Shedding light on the circadian clock and the photoperiodic control of flowering. *Curr. Opin. Plant Biol.* 6 13–19. 10.1016/s1369-5266(02)00011-0 12495746

[B17] Huang G. W. Ma J. H. Han Y. Z. Chen X. J. Fu Y. F. (2011). Cloning and expression analysis of the soybean CO-Like gene GmCOL9. *Plant Mol. Biol. Rep.* 29 352–359. 10.1007/s11105-010-0240-y

[B18] Imaizumi T. Schultz T. F. Harmon F. G. Ho L. A. Kay S. A. (2005). FKF1 F-box protein mediates cyclic degradation of a repressor of *CONSTANS* in *Arabidopsis*. *Science* 309, 293–297. 10.1126/science.1110586 16002617

[B19] Ito S. Song Y. H. Josephson-Day A. R. Miller R. J. Breton G. Olmstead R. G. (2012). FLOWERING BHLH transcriptional activators control expression of the photoperiodic flowering regulator CONSTANS in *Arabidopsis*. *Proc. Natl. Acad. Sci. U.S.A.* 109 3582–3587. 10.1073/pnas.1118876109 22334645 PMC3295255

[B20] Izawa T. (2021). What is going on with the hormonal control of flowering in plants? *Plant J.* 105 431–445. 10.1111/tpj.15036 33111430

[B21] Jang S. Marchal V. Panigrahi K. C. Wenkel S. Soppe W. Deng X. W. (2008). *Arabidopsis* COP1 shapes the temporal pattern of CO accumulation conferring a photoperiodic flowering response. *EMBO J.* 27 1277–1288. 10.1038/emboj.2008.68 18388858 PMC2291449

[B22] Koskela E. A. Mouhu K. Albani M. C. Kurokura T. Rantanen M. Sargent D. J. (2012). Mutation in TERMINAL FLOWER1 reverses the photoperiodic requirement for flowering in the wild strawberry *Fragaria vesca*. *Plant Physiol.* 159 1043–1054. 10.1104/pp.112.196659 22566495 PMC3387692

[B23] Kotake T. Takada S. Nakahigashi K. Ohto M. Goto K. (2003). *Arabidopsis* TERMINAL FLOWER 2 gene encodes a heterochromatin protein 1 homolog and represses both FLOWERING LOCUS T to regulate flowering time and several floral homeotic genes. *Plant Cell Physiol.* 44 555–564. 10.1093/pcp/pcg091 12826620

[B24] Kurokura T. Samad S. Koskela E. Mouhu K. Hytönen T. (2017). *Fragaria vesca* CONSTANS controls photoperiodic flowering and vegetative development. *J. Exp. Bot.* 68 4839–4850. 10.1093/jxb/erx301 29048562 PMC5853477

[B25] Lescot M. Déhais P. Thijs G. Marchal K. Moreau Y. Van de Peer Y. (2002). PlantCARE, a database of plant cis-acting regulatory elements and a portal to tools for in silico analysis of promoter sequences. *Nucleic Acids Res.* 30 325–327. 10.1093/nar/30.1.325 11752327 PMC99092

[B26] Liu J. H. Shen J. Q. Xu Y. Li X. H. Xiao J. H. Xiong L. Z. (2016). Ghd2, a CONSTANS-like gene, confers drought sensitivity through regulation of senescence in rice. *J. Exp. Bot.* 67 5785–5798. 10.1093/jxb/erw344 27638689 PMC5066496

[B27] Liu L. Ma J. Han Y. Chen X. Fu Y. F. (2011). The isolation and analysis of a soybean CO homologue GmCOL10. *Russ. J. Plant Physiol.* 58 330–336. 10.1134/S1021443711020105

[B28] Lu J. Sun J. Jiang A. Bai M. Fan C. Liu J. (2020). Alternate expression of CONSTANS-LIKE 4 in short days and CONSTANS in long days facilitates day-neutral response in *Rosa chinensis*. *J. Exp. Bot.* 71 4057–4068. 32227095 10.1093/jxb/eraa161 PMC7475255

[B29] Martin J. Storgaard M. Andersen C. H. Nielsen K. K. (2004). Photoperiodic regulation of flowering in perennial ryegrass involving a CONSTANS-like homolog. *Plant Mol. Biol.* 56 159–169. 10.1007/s11103-004-2647-z 15604735

[B30] Michaels S. D. Himelblau E. Sang Y. K. Amasino S. (2005). Integration of flowering signals in winter-annual *Arabidopsis*. *Plant Physiol.* 137 149–156. 10.1104/pp.104.052811 15618421 PMC548846

[B31] Moore R. C. Purugganan M. D. (2003). The early stages of duplicate gene evolution. *Proc. Natl. Acad. Sci. U.S.A.* 100 15682–15687. 10.1073/pnas.2535513100 14671323 PMC307628

[B32] Mouradov A. Crèmer F. Coupland G. (2002). Control of flowering time: interacting pathways as a basis for diversity. *Plant Cell* 14 111–130. 10.1105/tpc.001362 12045273 PMC151251

[B33] Pan G. Li Z. Yin M. Huang S. Q. Tao J. Chen A. G. (2021). Genome-wide identification, expression, and sequence analysis of CONSTANS-like gene family in cannabis reveals a potential role in plant flowering time regulation. *BMC Plant Biol.* 21:142. 10.1186/s12870-021-02913-x 33731002 PMC7972231

[B34] Perrella G. Vellutini E. Zioutopoulou A. Patitaki E. Headland L. R. Kaiserli E. (2020). Let it bloom: cross-talk between light and flowering signaling in *Arabidopsis*. *Physiol. Plant.* 169 301–311. 10.1111/ppl.13073 32053223 PMC7383826

[B35] Putterill J. Robson F. Lee K. Simon R. Coupland G. (1995). The CONSTANS gene of *Arabidopsis* promotes flowering and encodes a protein showing similarities to zinc-finger transcription factors. *Cell* 80 847–857. 7697715 10.1016/0092-8674(95)90288-0

[B36] Robson F. Costa M. M. Hepworth S. R. Vizir I. Pineiro M. Reeves P. H. (2001). Functional importance of conserved domains in the flowering-time gene CONSTANS demonstrated by analysis of mutant alleles and transgenic plants. *Plant J.* 28 619–631. 10.1046/j.1365-313x.2001.01163.x 11851908

[B37] Romero-Campero F. J. Lucas-Reina E. Said F. E. Romero J. M. Valverde F. (2013). A contribution to the study of plant development evolution based on gene co-expression networks. *Front. Plant Sci.* 4:291. 10.3389/fpls.2013.00291 PMC3732916 23935602

[B38] Saitou N. Nei M. (1987). The neighbor-joining method: a new method for reconstructing phylogenetic trees. *Mol. Biol. Evol.* 4 406–425. 10.1093/oxfordjournals.molbev.a040454 3447015

[B39] Samach A. Onouchi H. Gold S. E. Ditta G. S. Schwarz-Sommer Z. Yanofsky M. F. (2000). Distinct roles of CONSTANS target genes in reproductive development of *Arabidopsis*. *Science* 288 1613–1616. 10834834 10.1126/science.288.5471.1613

[B40] Sawa M. Kay S. A. (2011). GIGANTEA directly activates Flowering Locus T in *Arabidopsis thaliana*. *Proc. Natl. Acad. Sci. U.S.A.* 108 11698–11703. 10.1073/pnas.1106771108 21709243 PMC3136272

[B41] Song J. Irwin J. Dean C. (2013). Remembering the prolonged cold of winter. *Curr. Biol.* 23 807–811. 10.1016/j.cub.2013.07.027 24028964

[B42] Song X. M. Duan W. K. Huang Z. N. Liu G. F. Wu P. Liu T. K. (2015). Comprehensive analysis of the flowering genes in Chinese cabbage and examination of evolutionary pattern of CO-like genes in plant kingdom. *Sci. Rep.* 5:14631. 10.1038/srep14631 26416765 PMC4586889

[B43] Sønsteby A. Roos U. M. Heide O. M. (2017). Phenology, flowering and yield performance of 13 diverse strawberry cultivars grown under Nordic field conditions. *Acta. Agric. Scand Sect. B Soil Plant Sci.* 67 278–283.

[B44] Suárez-López P. Wheatley K. Robson F. Onouchi H. Valverde F. Coupland G. (2001). CONSTANS mediates between the circadian clock and the control of flowering in *Arabidopsis*. *Nature* 410 1116–1120. 10.1038/35074138 11323677

[B45] Talar U. Kiełbowicz-Matuk A. Czarnecka J. Rorat T. (2017). Genome-wide survey of B-box proteins in potato (*Solanum tuberosum*)-identification, characterization and expression patterns during diurnal cycle, etiolation and de-etiolation. *PLoS One* 12:e0177471. 10.1371/journal.pone.0177471 28552939 PMC5446133

[B46] Tamura K. Peterson D. Peterson N. Stecher G. Nei M. Kumar S. (2011). MEGA5: molecular evolutionary genetics analysis using maximum likelihood, evolutionary distance, and maximum parsimony methods. *Mol. Biol. Evol.* 28 2731–2739. 10.1093/molbev/msr121 21546353 PMC3203626

[B47] Tan J. J. Jin M. N. Wang J. C. Wu F. Q. Sheng P. K. Cheng Z. J. (2016). OsCOL10, a CONSTANS-Like gene, functions as a flowering time repressor downstream of Ghd7 in rice. *Plant Cell Physiol.* 57 798–812. 10.1093/pcp/pcw025 26872834

[B48] Tiwari S. B. Shen Y. Chang H. C. Hou Y. L. Harris A. Ma S. F. (2010). The flowering time regulator CONSTANS is recruited to the FLOWERING LOCUS T promoter via a unique cis-element. *New Phytol.* 187 57–66. 10.1111/j.1469-8137.2010.03251.x 20406410

[B49] Toledo-Ortiz G. Huq E. Quail P. H. (2003). The *Arabidopsis* basic/helix-loop-helix transcription factor family. *Plant Cell* 15 1749–1770. 10.1105/tpc.013839 12897250 PMC167167

[B50] Valverde F. (2011). CONSTANS and the evolutionary origin of photoperiodic timing of flowering. *J. Exp. Bot.* 62 2453–2463. 10.1093/jxb/erq449 21239381

[B51] Wang H. G. Zhang Z. L. Li H. Y. Zhao X. Y. Liu X. M. Ortiz M. (2013). CONSTANS-LIKE 7 regulates branching and shade avoidance response in *Arabidopsis*. *J. Exp. Bot.* 64 1017–1024. 10.1093/jxb/ers376 23314820 PMC3580813

[B52] Wang L. Xue J. Dai W. Tang Y. Gong P. Wang Y. (2019). Genome-wide identification, phylogenetic analysis, and expression profiling of CONSTANS-like (COL) genes in *Vitis vinifera*. *J. Plant Growth Regul.* 38 631–643. 10.1007/s00344-018-9878-8

[B53] Wenkel S. Turck F. Singer K. Gissot L. Le Gourrierec J. Samach A. (2006). Constans and the CCAAT box binding complex share a functionally important domain and interact to regulate flowering of *Arabidopsis*. *Plant Cell* 18 2971–2984. 10.1105/tpc.106.043299 17138697 PMC1693937

[B54] Wong A. C. Hecht V. F. Picard K. Diwadkar P. Laurie R. E. Wen J. (2014). Isolation and functional analysis of CONSTANS-LIKE genes suggests that a central role for CONSTANS in flowering time control is not evolutionarily conserved in *Medicago truncatula*. *Front. Plant Sci.* 5:486. 10.3389/fpls.2014.00486 25278955 PMC4166892

[B55] Wu W. X. Zheng X. M. Chen D. B. Zhang Y. X. Ma W. W. Zhang H. (2017). OsCOL16, encoding a CONSTANS-like protein, represses flowering by up-regulating Ghd7 expression in rice. *Plant Sci.* 260 60–69. 10.1016/j.plantsci.2017.04.004 28554475

[B56] Yang N. Cong Q. Cheng L. J. (2019). BBX transcriptional factors family in plants a review. *Chin. J. Biotech.* 36 666–677. 10.13345/j.cjb.190302 32347061

[B57] Yang T. He Y. Niu S. Yan S. Zhang Y. (2020). Identification and characterization of the CONSTANS (CO)/CONSTANS-like (COL) genes related to photoperiodic signaling and flowering in tomato. *Plant Sci.* 301:110653. 10.1016/j.plantsci.2020.110653 33218623

[B58] Yano M. Katayose Y. Ashikari M. Yamanouchi U. Monna L. Fuse T. (2000). Hd1, a major photoperiod sensitivity quantitative trait locus in rice, is closely related to the *Arabidopsis* flowering time gene CONSTANS. *Plant Cell* 12 2473–2484. 10.1105/tpc.12.12.2473 11148291 PMC102231

[B59] Yu J. Wang J. Lin W. Li S. Li H. Zhou J. (2005). The genomes of *Oryza sativa*: a history of duplications. *PLoS Biol.* 3:e38. 10.1371/journal.pbio.0030038 PMC546038 15685292

[B60] Zhu Y. Klasfeld S. Jeong C. W. Jin R. Goto K. Yamaguchi N. (2020). TERMINAL FLOWER 1-FD complex target genes and competition with FLOWERING LOCUS T. *Nat. Commun.* 11:5118. 10.1038/s41467-020-18782-1 33046692 PMC7550357

