# Supplementary material for: Genome-Wide Identification, Characterization, and Expression Profile Analysis of CONSTANS-like Genes in Woodland Strawberry (Fragaria vesca)
Source: Front Plant Sci. 2022 Jul 12;13:931721. doi: 10.3389/fpls.2022.931721 (PMC9318167; doi:10.3389/fpls.2022.931721)
Supplement: Supplementary file 1 [file Data_Sheet_1.docx]

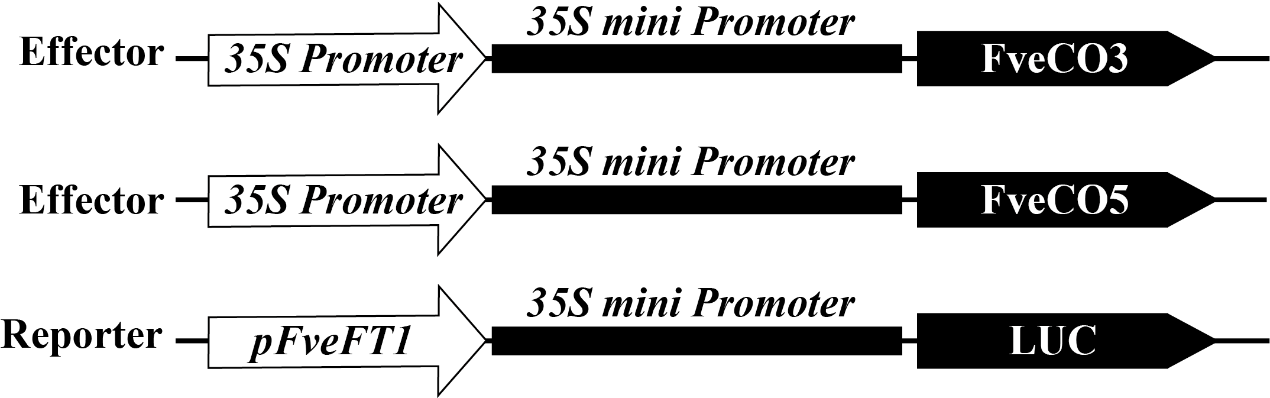


**Supplementary** **Figure S1∣**Schematic diagram of vector construction in luciferase reporter analysis.


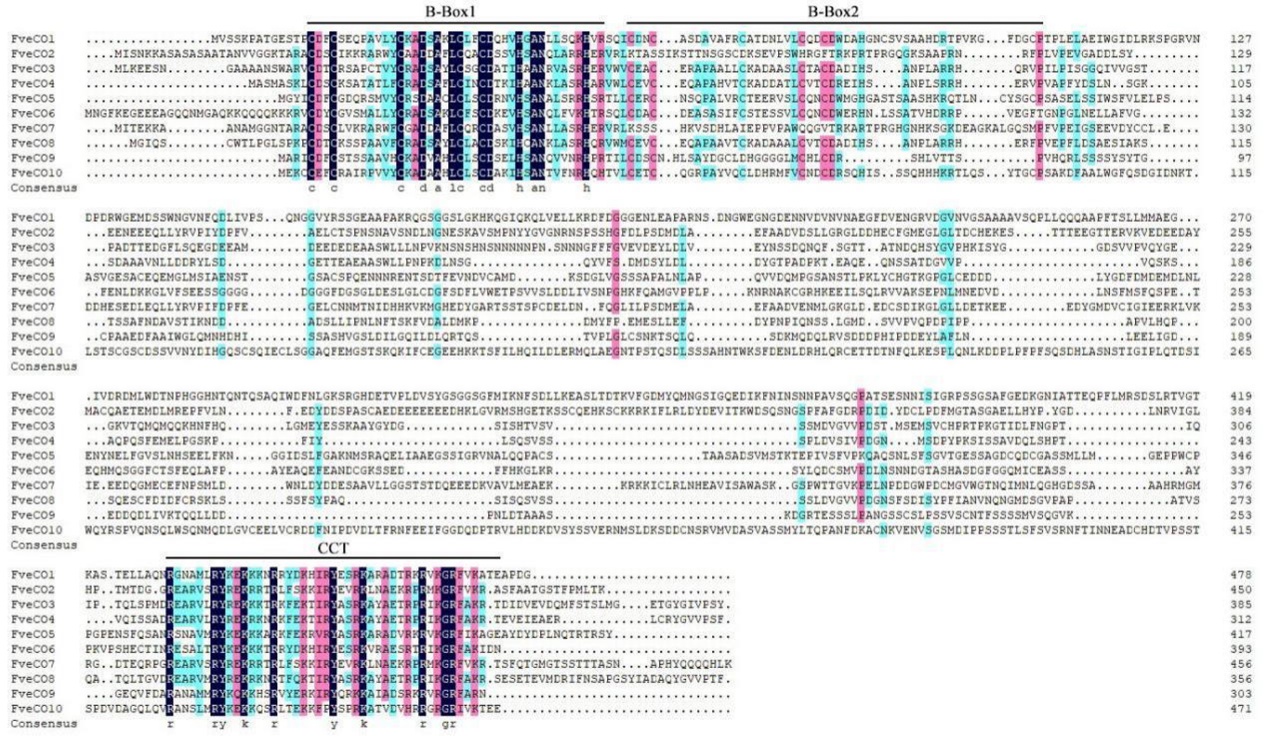


**Supplementary** **Figure S2∣**Sequence alignment of woodland strawberry CO-like proteins. The CO family signature in FveCOs is marked with the domain names. The conserved residues are indicated by a colored background.


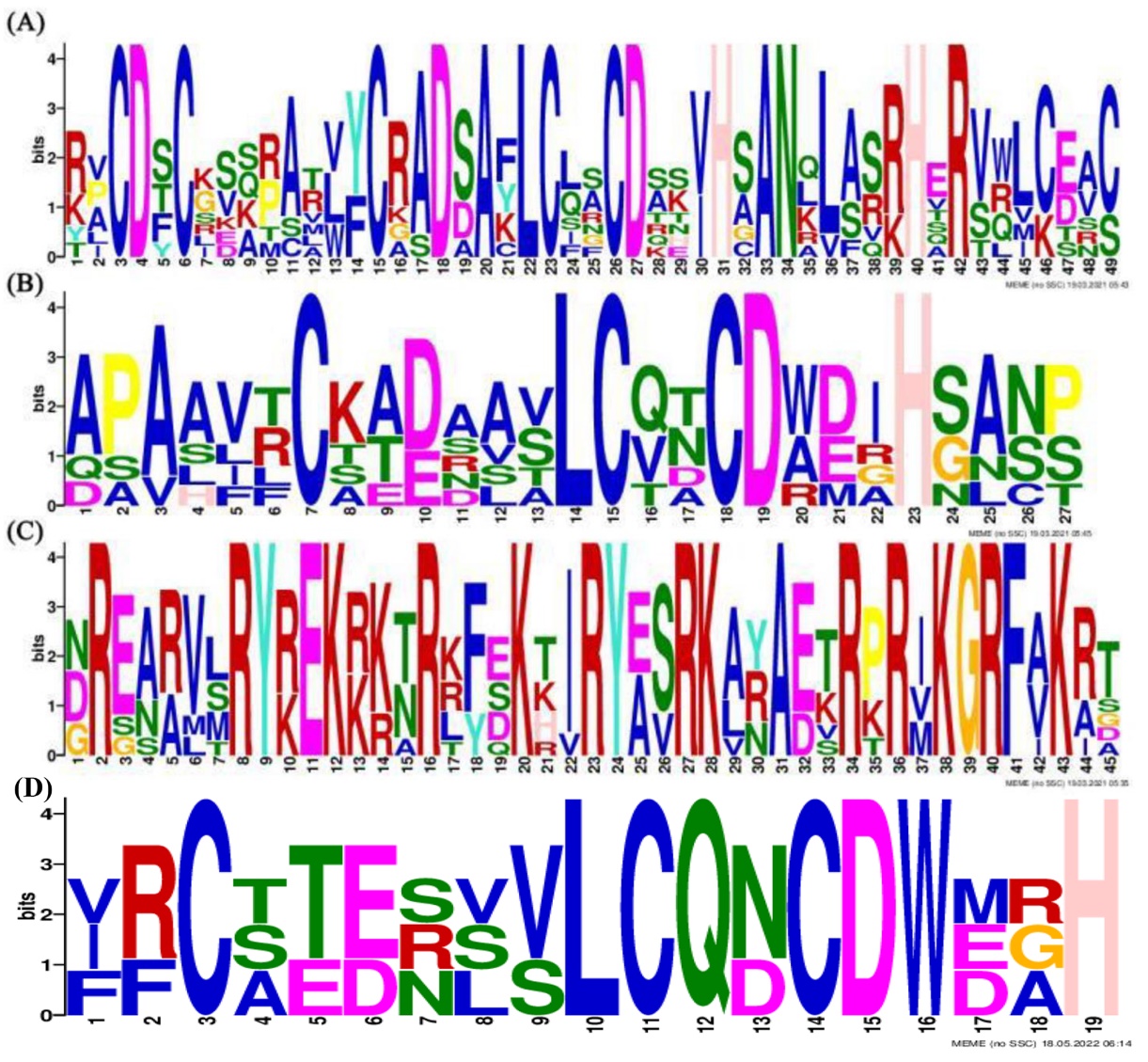


**Supplementary** **Figure S3∣**Sequence logo analyses of conserved amino acid residues in several domains of FveCOs proteins. (A) Conserved amino acid residues in B-box1 domain; (B) Conserved amino acid residues in B-box 2 domain; (C) Conserved amino acid residues in CCT domain; (D) Conserved amino acid residues in DZF domain. The logos of each domain were generated from the alignment of 10 FveCOs sequences by the MEME software. The font size represents the conservation level of domains. The larger the font size, the higher the conservation.
