# Supplementary material for: Genome-Wide Identification, Characterization, and Expression Profile Analysis of CONSTANS-like Genes in Woodland Strawberry (Fragaria vesca)
Source: Front Plant Sci. 2022 Jul 12;13:931721. doi: 10.3389/fpls.2022.931721 (PMC9318167; doi:10.3389/fpls.2022.931721)
Supplement: Supplementary file 2 [file Data_Sheet_2.docx]

| **Gene name** | **Primer F** | **Primer R** |
| --- | --- | --- |
| *FveCO1* | AAGCAGGGAATTCAGAAGCA | TTCCGCATTCACATTCACAT |
| *FveCO2* | CTCCGTTTGCATTTGGAGAT | AGCCTCGTCCTCCTCTTTTC |
| *FveCO3* | CCAAGAACACCGAAAGGAAC | CTTGAGGCATACCGGATTGT |
| *FveCO4* | TACCCCAAGTCGATCAGCTC | CCTTTGATTCGTGGTCTGGT |
| *FveCO5* | TGCATTCTGCTAATGCCTTG | TGAAGCAGCTGAAGTGGATG |
| *FveCO6* | AACCGGAATGCTAAGTGTGG | AGCAGGAAATGCAAGCTGTT |
| *FveCO7* | GCTGAATCATGAGGCAGTCA | TATCGCCTCTCATTCCCATC |
| *FveCO8* | TGGGTATGGATAGCGTGGTT | CTAAGGAGGACGACGAGACG |
| *FveCO9* | ATGCAACCACCTCTCAGCTT | TCCCCACATGACTTGCACTA |
| *FveCO10* | TCCGGTCCAAAATAGTCAGC | TCATGCAGCACTCTGGTAGG |
| *pRI101-FveCO3* | TCTTCACTGTTGATACATATGTTGAAAGAAGAGAGCAATGGC | CGATCGGGGAAATTCGAGCTCTTAGTATGAAGGAACAATGCCGTATC |
| *pRI101-FveCO5* | TCTTCACTGTTGATACATATGGGTTACATATGCGACTTC | CGATCGGGGAAATTCGAGCTCTCAGTAGCTTCTGGTTCTGGT |
| *LUC-proFveFT1* | AGATCGAATTCCATGGCTTATTTCAAGTGTATATGTC | TTGGCGTCTTCCATGGATTGATCCTTCAAGCTAGCTAG |

**Table S1 Primers used in this study**

**Supplemental Table S2. CREs identified in promoters of *FveCOs*.**

| **Site name** | **Number** | **Sequence** | **Function of the cis-elements** |
| --- | --- | --- | --- |
| TATA-box | 283 | TATA | core promoter element around -30 of transcription start |
| G-box | 23 | CACGTG | cis-acting regulatory element involved in light responsiveness |
| CAT-box | 7 | GCCACT | cis-acting regulatory element related to meristem expression |
| MBS | 6 | CAACTG | MYB binding site involved in drought-inducibility |
| TCA-element | 6 | CCATCTTTTT | cis-acting element involved in salicylic acid responsiveness |
| AT-rich element | 3 | ATAGAAATCAA | binding site of AT-rich DNA binding protein (ATBP-1) |
| TGACG-motif | 17 | TGACG | cis-acting regulatory element involved in the MeJA-responsiveness |
| CAAT-box | 109 | CCAAT | common cis-acting element in promoter and enhancer regions |
| O2-site | 8 | GTTGACGTGA | cis-acting regulatory element involved in zein metabolism regulation |
| CGTCA-motif | 17 | CGTCA | cis-acting regulatory element involved in the MeJA-responsiveness |
| TGA-element | 7 | AACGAC | auxin-responsive element |
| ARE | 16 | AAACCA | cis-acting regulatory element essential for the anaerobic induction |
| HD-Zip 1 | 2 | CAAT(A/T)ATTG | element involved in differentiation of the palisade mesophyll cells |
| A-box | 4 | CCGTCC | cis-acting regulatory element |
| GA-motif | 1 | ATAGATAA | part of a light responsive element |
| ABRE | 21 | CACGTG | cis-acting element involved in the abscisic acid responsiveness |
| GCN4_motif | 1 | TGAGTCA | cis-regulatory element involved in endosperm expression |
| MRE | 5 | AACCTAA | MYB binding site involved in light responsiveness |
| P-box | 4 | CCTTTTG | gibberellin-responsive element |
| Unnamed__1 | 1 | GAATTTAATTAA | 60K protein binding site |
| MBSI | 1 | aaaAaaC(G/C)GTTA | MYB binding site involved in flavonoid biosynthetic genes regulation |
| GT1-motif | 16 | GGTTAA | light responsive element |
| AE-box | 7 | AGAAACTT | part of a module for light response |
| LS7 | 1 | CAGATTTATTTTTA | part of a light responsive element |
| Box 4 | 8 | ATTAAT | part of a conserved DNA module involved in light responsiveness |
| Sp1 | 1 | GGGCGG | light responsive element |
| TCCC-motif | 4 | TCTCCCT | part of a light responsive element |
| LTR | 5 | CCGAAA | cis-acting element involved in low-temperature responsiveness |
| GARE-motif | 3 | TCTGTTG | gibberellin-responsive element |
| Gap-box | 1 | CAAATGAA(A/G)A | part of a light responsive element |
| GATA-motif | 7 | AAGATAAGATT | part of a light responsive element |
| I-box | 3 | AAGATAAGGCT | part of a light responsive element |
| ATC-motif | 2 | AGTAATCT | part of a conserved DNA module involved in light responsiveness |
| RY-element | 2 | CATGCATG | cis-acting regulatory element involved in seed-specific regulation |
| TATC-box | 1 | TATCCCA | cis-acting element involved in gibberellin-responsiveness |
| chs-CMA2c | 1 | ATGTACGTGGAGG | part of a light responsive element |
| ATCT-motif | 1 | AATCTAATCC | part of a conserved DNA module involved in light responsiveness |
| TCT-motif | 5 | TCTTAC | part of a light responsive element |
| CCAAT-box | 1 | CAACGG | MYBHv1 binding site |
| AuxRR-core | 1 | GGTCCAT | cis-acting regulatory element involved in auxin responsiveness |
| LAMP-element | 2 | CTTTATCA | part of a light responsive element |
| chs-CMA1a | 1 | TTACTTAA | part of a light responsive element |
| TC-rich repeats | 1 | GTTTTCTTAC | cis-acting element involved in defense and stress responsiveness |

**Supplementary Table S3. Accession numbers of *CO*/*COL* orthologs used in phylogenetic tree.**

| **Gene name** | **Gene ID** | **Gene name** | **Gene ID** | **Gene name** | **Gene ID** |
| --- | --- | --- | --- | --- | --- |
| *FveCO1* | *FvH4_7g05240.1* | *StCOL13* | *PGSC0003DMP400020149* | *ZmCO5* | *ACG28357* |
| *FveCO2* | *FvH4_4g27390.1* | *StCOL14* | *PGSC0003DMP400044086* | *ZmCO6* | *ACG30244* |
| *FveCO3* | *FvH4_6g45860.1* | *StCOLX* | *PGSC0003DMP400050198* | *ZmCO8* | *ACG30381* |
| *FveCO4* | *FvH4_2g41420.1* | *StCOL16a* | *PGSC0003DMP400025662* | *ZmCO10* | *PWZ05768* |
| *FveCO5* | *FvH4_6g40380.1* | *StCOL16b* | *PGSC0003DMP400013670* | *ZmCO16* | *ACG35514* |
| *FveCO6* | *FvH4_2g24910.1* | *StCOL16c* | *PGSC0003DMP400009918* | *SbCOL3* | *XP_021313837* |
| *FveCO7* | *FvH4_5g12150.1* | *NtCOL2* | *NP_001311813* | *SbCOL4* | *XP_002446734* |
| *FveCO8* | *FvH4_4g08980.1* | *NtCOL4* | *XP_016509100* | *SbCOL5* | *XP_002438778* |
| *FveCO9* | *FvH4_4g26540.1* | *NtCOL5* | *XP_016435645* | *SbCOL9* | *XP_002454366* |
| *FveCO10* | *FvH4_4g26550.1* | *NtCOL6* | *XP_016478271* | *SbCOL13* | *XP_002463352* |
| *AtCO* | [*At5G15840*](http://arabidopsis.org/servlets/TairObject?type=gene&name=At5G15840.1) | *NtCOL9* | *XP_016497584* | *SbCOL15* | *XP_021314480* |
| *AtCOL1* | [*AT5G15850*](https://solgenomics.net/tools/blast/match/show?blast_db_id=8;id=AT5G15850.1;hilite_coords=1-355) | *NtCOL10* | *XP_016497773* | *SbCOL16* | *XP_002466587* |
| *AtCOL2* | [*AT3G02380*](https://solgenomics.net/tools/blast/match/show?blast_db_id=8;id=AT3G02380.1;hilite_coords=1-347) | *NtCOL13* | *XP_016509923* | *VvCOL4* | *VIT_04s0008g07340* |
| *AtCOL3* | [*AT2G24790*](https://solgenomics.net/tools/blast/match/show?blast_db_id=8;id=AT2G24790.1;hilite_coords=1-294) | *NtCOL14* | *XP_016501683* | *VvCOL5* | *VIT_11s0052g01800* |
| *AtCOL4* | [*AT5G24930*](https://solgenomics.net/tools/blast/match/show?blast_db_id=8;id=AT5G24930.1;hilite_coords=45-406) | *NtCOL16a* | *XP_016453930* | *VvCOL9a* | *VIT_00s0194g00070* |
| *AtCOL5* | [*AT5G57660*](https://solgenomics.net/tools/blast/match/show?blast_db_id=8;id=AT5G57660.1;hilite_coords=1-355) | *NtCOL16b* | *XP_016434941* | *VvCOL9b* | *VIT_12s0057g01350* |
| *AtCOL6* | [*AT1G68520*](https://solgenomics.net/tools/blast/match/show?blast_db_id=8;id=AT1G68520.1;hilite_coords=1-406) | *OsHd1/OsA* | *LOC_Os06g16370* | *VvCOL11a* | *VIT_12s0059g02500* |
| *AtCOL7* | *AT1G73870* | *OsB* | *LOC_Os09g06464* | *VvCOL11b* | *VIT_19s0014g05120* |
| *AtCOL8* | *AT1G49130* | *OsC* | *LOC_Os04g42020* | *VvCOL13* | *VIT_07s0104g01360* |
| *AtCOL9* | *AT3G07650* | *OsD* | *LOC_Os02g39710* | *VvCOL14a* | *VIT_14s0068g01380* |
| *AtCOL10* | *AT5G48250* | *OsE* | *LOC_Os06g44450* | *VvCOL14b* | *VIT_01s0146g00360* |
| *AtCOL11* | *AT4G15250* | *OsF* | *LOC_Os02g08150* | *VvCOL16a* | *VIT_01s0011g03520* |
| *AtCOL12* | *AT3G21880* | *OsG* | *LOC_Os08g15050* | *VvCOL16b* | *VIT_17s0000g03740* |
| *AtCOL13* | *AT2G47890* | *OsJ* | *LOC_Os03g50310* | *PaCOL1* | *EU815826* |
| *AtCOL14* | *AT2G33500* | *OsK* | *LOC_Os02g49880* | *PaCOL2* | *EF650085* |
| *AtCOL15* | *AT1G28050* | *OsL* | *LOC_Os06g15330* | *PdCO* | *AAS00054* |
| *AtCOL16* | *AT1G25440* | *OsM* | *LOC_Os06g19444* | *PdCO2* | *AAS00055* |
| *SlCO1* | *Solyc02g089540* | *OsN* | *LOC_Os02g49230* |  |  |
| *SlCO3* | *Solyc02g089520* | *OsO* | *LOC_Os08g42440* |  |  |
| *SlCOL* | *Solyc07g006630* | *OsP* | *LOC_Os03g22770* |  |  |
| *SlCOL4a* | *Solyc12g096500* | *BvCOL1* | *ACC95129* |  |  |
| *SlCOL4b* | *Solyc08g006530* | *BvCOL2* | *ACC95129* |  |  |
| *SlCOL10a* | *Solyc07g045180* | *BvCOL3* | *ACC95131* |  |  |
| *SlCOL10b* | *Solyc05g020020* | *CrCO* | *CAP74566* |  |  |
| *SlCOL12* | *Solyc05g046040* | *GmCOL2* | *ABD28283* |  |  |
| *SlCOL13* | *Solyc09g074560* | *GmCOL4* | *XP_003526113* |  |  |
| *SlCOL14* | *Solyc05g024010* | *GmCOL5* | *NP_001240941* |  |  |
| *SlCOL16a* | *Solyc04g007210* | *GmCOL9* | *XP_014622613* |  |  |
| *SlCOL16b* | *Solyc03g119540* | *GmCOL16* | *XP_003555902* |  |  |
| *SlCOL16c* | *Solyc05g009310* | *LpCO* | *CAH55695* |  |  |
| *StCO* | *PGSC0003DMP400017796* | *LeCOL1* | *AAS67376* |  |  |
| *StCOL2* | *PGSC0003DMP400017799* | *LeCOL2* | *AAS67378* |  |  |
| *StCOL5* | *PGSC0003DMP400051145* | *LeCOL3* | *AAS67379* |  |  |
| *StCOL4a* | *PGSC0003DMP400045675* | *TaHd1* | *BAC92736* |  |  |
| *StCOL4b* | *PGSC0003DMP400047781* | *HvCO1* | *AAM74062* |  |  |
| *StCOL10a* | *PGSC0003DMP400030431* | *MdCO1* | *ACD69427* |  |  |
| *StCOL10b* | *PGSC0003DMP400009430* | *MdCO2* | *ACD69428* |  |  |
| *StCOL12* | *PGSC0003DMP400002288* | *PnCO* | *AAG24863* |  |  |
