# Supplementary material for: Genome-Wide Identification, Characterization, and Expression Profile Analysis of CONSTANS-like Genes in Woodland Strawberry (Fragaria vesca)
Source: Front Plant Sci. 2022 Jul 12;13:931721. doi: 10.3389/fpls.2022.931721 (PMC9318167; doi:10.3389/fpls.2022.931721)
Supplement: Supplementary file 3 [file Data_Sheet_3.docx]

Input files for protein interaction networks prediction of FveCO3 and FveCO5

>FveCO3

MLKEESNGAAAANSWARVCDTCRSAPCTVYCRADSAYLCSGCDATIHAANRVASRHERVWVCEACERAPAALLCKADAASLCTACDADIHSANPLARRHQRVPILPISGGQIVVGSTPADTTEDGFLSQEGDEEAMDEEDEDEAASWLLLNPVKNSNSHNSNNNNNPNSNNNGFFFGVEVDEYLDLVEYNSSDQNQFSGTTATNDQHSYGVPHKISYGGDSVVPVQYGEGKVTQMQMQQKHNFHQLGMEYESSKAAYGYDGSISHTVSVSSMDVGVVPDSTMSEMSVCHPRTPKGTIDLFNGPTIQIPTQLSPMDREARVLRYREKKKTRKFEKTIRYASRKAYAETRPRIKGRFAKRTDIDVEVDQMFSTSLMGETGYGIVPSY

>FveCO5

MGYICDFCGDQRSMVYCRSDAACLCLSCDRNVHSANALSRRHSRTLLCERCNSQPALVRCTEERVSLCQNCDWMGHGASTSAASHKRQTLNCYSGCPSASELSSIWSFVLELPSASVGESACEQEMGLMSIAENSTGSACSPQENNNRENTSDTFEVNDVCAMDKSDGLVGSSSAPALNLAPQVVDQMPGSANSTLPKLYCHGTKGPGLCEDDDLYGDFDMDEMDLNLENYNELFGVSLNHSEELFKNGGIDSLFGAKNMSRAQELIAAEGSSIGRVNALQQPACSTAASADSVMSTKTEPIVSFVPKQAQSNLSFSGVTGESSAGDCQDCGASSMLLMGEPPWCPPGPENSFQSANRSNAVMRYKEKKKARKFEKRVRYASRKARADVRKRVKGRFIKAGEAYDYDPLNQTRTRSY
